# Enhancing Time-Series Detection Algorithms for Automated Biosurveillance

**DOI:** 10.3201/1504.080616

**Published:** 2009-04

**Authors:** Jerome I. Tokars, Howard Burkom, Jian Xing, Roseanne English, Steven Bloom, Kenneth Cox, Julie A. Pavlin

**Affiliations:** Centers for Disease Control and Prevention, Atlanta, Georgia, USA (J.I. Tokars, J. Xing, R. English); The Johns Hopkins University, Baltimore, Maryland, USA (H. Burkom); Science Applications Incorporated, San Diego, California, USA (S. Bloom); Department of Defense, Washington, DC, USA (K. Cox); Armed Forces Research Institute of Medical Sciences, Bangkok, Thailand (J.A. Pavlin)

**Keywords:** Surveillance, algorithms, statistics, data analysis, outbreak detection, research

## Abstract

BioSense is a US national system that uses data from health information systems for automated disease surveillance. We studied 4 time-series algorithm modifications designed to improve sensitivity for detecting artificially added data. To test these modified algorithms, we used reports of daily syndrome visits from 308 Department of Defense (DoD) facilities and 340 hospital emergency departments (EDs). At a constant alert rate of 1%, sensitivity was improved for both datasets by using a minimum standard deviation (SD) of 1.0, a 14–28 day baseline duration for calculating mean and SD, and an adjustment for total clinic visits as a surrogate denominator. Stratifying baseline days into weekdays versus weekends to account for day-of-week effects increased sensitivity for the DoD data but not for the ED data. These enhanced methods may increase sensitivity without increasing the alert rate and may improve the ability to detect outbreaks by using automated surveillance system data.

## Abstract 2

Algorithm modifications may improve sensitivity for detecting artificially added data.

Since the late 1990s, the threats of bioterrorist attacks, the potential for outbreaks of natural disease such as severe acute respiratory syndrome and pandemic influenza, and the availability of computerized data have prompted the use of automated disease surveillance systems (*1*). Sources of information include clinical data, such as records of hospital emergency department visits, and nonclinical information, such as sales of over-the-counter remedies (*2*). However, human resources are limited for interpreting the large volume of available information. Thus, statistical algorithms are needed to filter large volumes of data, focus attention on potential public health problems, and provide an objective measure of increases in disease activity.

BioSense is a US national automated surveillance system that receives data from various sources and makes them available for public health use. The data may be viewed simultaneously by local, state, and federal public health officials through the Internet-based BioSense Application, which may be accessed on a jurisdiction-specific basis through the Centers for Disease Control and Prevention (CDC) Secure Data Network (*3*). Data received include coded final diagnoses and free-text chief complaints, which are assigned as appropriate to >1 of 11 syndrome groupings representing general illness categories such as respiratory and gastrointestinal illnesses (*4*) and to >1 of 78 subsyndromes representing more specific categories such as asthma or cough (*5*). To identify days when disease indicator activity is higher than expected, BioSense uses a modified version of the C2 algorithm, 1 of 3 algorithms (C1, C2, and C3) developed for the Early Aberration Reporting System (EARS) (*6**,**7*).

The C2 algorithm uses a sliding baseline of 7 consecutive recent days’ counts to calculate a mean (µ) and SD (s_t_). The test statistic is (x_t_ – µ)/s_t_, the number of SDs by which the current value x_t_ exceeds µ, or 0 if x_t_ does not exceed µ. EARS uses a test statistic >3 to signal an alert (*6**,**7*). Owing to their simplicity, ease of implementation, and implicit correction for seasonal trends (only data from the prior 9 days are used), the EARS algorithms are widely used (*8**–**10*). However, the algorithms do not perform optimally under all circumstances. First, because daily counts often vary by day of week, many alerts may be produced on high-count days such as Mondays and Tuesdays, and few may be produced on low-count days such as weekend days. Second, the short (7-day) baseline period may produce unstable values for the mean and SD; thus, the minimum daily count that triggers an alert may vary widely over a short period. Third, using simple count data does not account for the population at risk, which is generally unknown in these systems and which may vary, especially during crisis situations. Although C2 can be used on rates rather than counts, prior evaluations have not shown that using rates improves performance (L. Hutwagner, pers. comm.). Finally, occurrences of many disease indicators are rare, resulting in calculations for both expected values and SDs of 0; the EARS methods are not recommended in such instances. A minimum SD may be used to avoid division by zero, but if this minimum value is set to 0.2, a count of 1 will be 5 SDs above the mean and trigger a high-level alert.

This article describes and evaluates modifications of C2 that retain its inherent advantages, address its potential limitations, and improve its performance. We used real daily syndrome counts from 2 sources as baseline data and assessed the ability of various algorithms to detect additional counts artificially added to the data. Because all analyses were conducted at a constant alert rate of 1%, improvements in sensitivity were not accompanied by an increase in alerts.

## Methods

Four algorithm modifications, designed to address shortcomings in the C2 algorithm, were tested. The first modification tested was stratification by weekdays versus weekend days. Although many methods have been used to adjust for differing counts by day of week (*11*), these methods may require customization to specific datasets and a long data history (up to several years). Our simple method is to stratify the baseline days used to calculate µ and s_t_ into weekdays versus weekend days. This stratification is denoted the W2 algorithm. For example, a 7-day W2 baseline for weekdays contains the most recent 7 weekdays. For unstratified and stratified analyses, the 2 days immediately before the index day were excluded from the baseline, a standard practice for C2, to avoid contamination with the upswing of an outbreak.

The second modification tested was lengthening the baseline period. Because a 7-day period may provide insufficient data for an accurate and stable calculation of µ and s_t_, we tested baseline periods of 7, 14, and 28 days. However, because we used data from <56 days before the index day, the stratified 28-day baseline will include only ≈16 days for weekend days.

The third modification tested was adjustment for total daily visits. For the adjustment procedure, we used a formula in which n_0_ = count of visits on the index day for the chosen syndrome (e.g., visits for the respiratory syndrome), and d_0_ = the total number of facility visits on the index day, including visits that were both assigned and unassigned to any of the 11 syndromes. Σn_i_ = total syndrome visits summed for all i baseline days. Σd_i_ = total facility visits summed for all i baseline days. The formula for the adjusted expected value was e_0_ = d_0_ × Σn_i_/Σd_i_, which differed considerably from the mean of the n_i_ if d_0_ was high or low. Fewer visits for a given syndrome were thus expected on a day when the facility had fewer total visits. The estimated adjusted SD, s_0_, was taken as the mean absolute value of (n_i_ – d_i_ × Σn_i_/Σd_i_) over i baseline days; that is, s_0_ = Σ (abs(n_i_ – d_i_ × Σn_i_/Σd_i_))/i. The test statistic adjusted for total visits was (n_0_ – e_0_)/s_0_, analogous to the C2 statistic (n_0_ – µ)/s_t_, where µ and s_t_ are the mean and SD of n_i_, the counts on baseline days. In the discussion below, we refer to this adjustment as the rate algorithm.

The fourth modification tested was increased minimum value for SD. We studied minimum values of 0.2 and 1.0.

To test these modifications, 2 datasets were used: records of Department of Defense (DoD) facility final diagnoses for September 2004–November 2007 and records of hospital emergency department (ED) chief complaints for March 2006–November 2007. The DoD data consisted primarily of data from outpatient clinics; however, ≈15% of the visits in this evaluation were from patients seen in emergency facilities and cannot currently be differentiated in the BioSense System. We studied the 11 syndrome groups designed to be indicative of infections resulting from exposure to pathogens plausibly used in a bioterrorist attack (*4*). The DoD data consisted of daily counts of patient visits with International Classification of Diseases, 9th Revision (ICD-9)–coded diagnoses categorized into the 11 syndrome groups. The hospital ED data consisted of free-text chief complaints, which were first parsed for a specified set of keywords, abbreviations, and misspellings and then categorized into 10 of the syndrome groups (1 syndrome, specific infection, was used for diagnosis but not for chief complaint data). Some ICD-9 codes and chief complaints may be included in >2 syndromes. However, counts of different syndromes were analyzed separately, not added together, and therefore are not double-counted in the analyses. For both datasets, we analyzed counts aggregated by facility. We included facility-syndrome combinations that had mean counts >0.5 over all facility–syndrome days in the study period. Many DoD clinics are closed on holidays. Therefore, for the DoD data, 11 days (days on which federal holidays are observed and the day after Thanksgiving) were recoded as weekend days for purposes of stratified algorithm calculations (*5*). Because hospital EDs typically are open on these holidays, no recoding for holidays was performed for this dataset.

The mean count for each facility syndrome was calculated and categorized as follows: 0.5 to <2, 2 to <4, 4 to <6, 6 to <8, 8 to <10, 10 to <20, 20 to <40, and >40. Empirical distributions of the test statistic (e.g., number of SDs by which the observed count exceeds the expected value) were conducted separately for each dataset, algorithm, and mean count category; the 99th percentile value for each of these distributions was used as the cutoff value to define an alert rate of 1%. For example, for the standard C2 algorithm in DoD data with mean count 4 to <6, a cutoff value of 3.9 was used because 1% of the facility-syndrome days had a test statistic >3.9. Because no attempt was made to find and exclude real outbreaks from the data, these cutoff values define an alert rate rather than a false alert rate, the latter being equivalent to 1-specificity (*12*).

At a constant alert rate of 1% for all methods, the sensitivity for detecting additional counts was calculated by performing the following steps: 1) running the algorithm to determine expected values and SDs for each facility-syndrome-day; 2) finding the 99th percentile cutoff value for the test statistic for each dataset-algorithm-mean count category as explained above; 3) for each facility-syndrome day, determining whether the observed count plus additional counts is greater than or equal to the threshold value (threshold value = expected value + SD × 99th percentile cutoff value); and 4) calculating sensitivity as the percentage of days on which the additional counts would exceed the threshold value and therefore be detected. Using this method, a single computer run can calculate sensitivity for detecting single-day additional counts on all days in the dataset; if the additional counts are spread over multiple days, separate computer runs would be needed (*7*).

## Results

The DoD diagnosis data contained 1,939,993 facility–syndrome days from 308 facilities in 48 states with an overall mean of 7.7 counts per facility per day; of the 11 syndromes, respiratory visits comprised the highest percentage (16% of total facility–syndrome days) and had the highest mean count (26.0 visits per facility per day) (Table 1). The hospital ED data contained 768,195 facility–syndrome days from 340 facilities in 21 states and had an overall mean of 7.8 counts per facility per day; no visits for lymphadenitis and severe injury and death were included because no facilities had a mean count >0.5 per day for these syndromes.

**Table 1:** Distribution of hospital emergency department visits and mean count per day, by syndrome and dataset, for selected BioSense data used in algorithm modification study*

Syndrome	Department of Defense clinic diagnosis			Hospital emergency department chief complaint	
	Mean count/d	% Facility–syndrome days		Mean count/d	% Facility–syndrome days
Botulism-like	2.5	3.8		0.9	1.8
Fever	4.4	10.1		6.3	14.3
Gastrointestinal	8.9	13.7		14.5	14.7
Hemorrhagic	2.2	5.7		2.6	13.6
Localized cutaneous lesion	3.0	10.8		2.6	13.2
Lymphadenitits	1.1	4.8		NA	0†
Neurologic	3.6	10.6		5.2	14.4
Rash	4.3	11.2		2.2	13.1
Respiratory	26.0	16.0		20.0	14.7
Severe injury and death	2.2	2.6		NA	0†
Specific infection	3.2	10.7		NA‡	0‡
All	7.7	100		7.8	100

*NA, not applicable. †Facilities were not included because none had mean counts >0.5 for syndromes. ‡Chief complaint data are not assigned to this syndrome.

**Figure 1:**
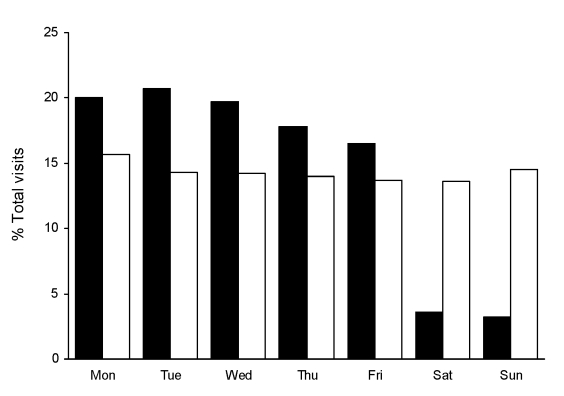
Distribution of syndrome counts, by day of week and data source, for selected BioSense data used in algorithm modification study. Black bars show Department of Defense data, and white bars show hospital emergency department data.

The DoD data had a strong day-of-week effect; 16%–21% of total weekly visits occurred per day on weekdays, and only 3%–4% of visits occurred per day on weekend days and holidays (Figure 1). The hospital ED data had a minimal day-of-week effect: 14%–16% of visits occurred per day on weekdays, and 14%–15% of visits occurred per day on weekend days.

The accuracy of expected value calculation was evaluated by using mean absolute residuals. For lower residuals, expected values are closer to observed values than they are for higher residuals. Similarly, the expected value calculation is more accurate for lower residuals than for higher residuals. For the DoD data, lower residuals were seen with stratification (W2) and the rate algorithm: mean residual 4.2 for unstratified count algorithm versus 2.2 for stratified rate algorithm (Table 2). For the hospital ED data, residuals were lower for the rate algorithm, and stratification had a minimal effect. Varying the baseline duration and minimum SD had no effect on the accuracy of expected value calculation (data not shown).

**Table 2:** Mean absolute residual, by method and dataset, for selected BioSense data used in algorithm modification study*

Stratification of baseline by weekday vs. weekend	Mean absolute residual				
	Department of Defense			Hospital emergency department	
	Count	Rate		Count	Rate
Unstratified	4.2	2.4		2.2	2.0
Stratified	2.4	2.2		2.3	2.0

*The count method uses only numerator data; the rate method uses numerator and denominator data. Because varying the baseline duration did not affect residuals (data not shown), all calculations shown here use a baseline duration of 7 days.

The effect of modifications of the initial algorithm on the sensitivity for detecting additional counts was examined; each modification was added consecutively (Table 3). For the DoD data, sensitivity was 40.6% for the initial algorithm and increased to 43.9% when the rate method was used; 70.8% when the minimum SD was increased to 1.0; 79.4% when the baseline duration was increased to 28 days; and 82.0% when a stratified baseline was used. Comparing the initial algoithm to the best algorithm showed a 41.4% increase in sensitivity. For the hospital ED data, sensitivity was 40.2% for the initial algorithm and increased to 64.8% for the best method (minimum SD = 1, 28-day baseline, rate method, unstratified baseline); however, when the stratified baseline was used, sensitivity decreased to 62.1%; the initial algorithm compared with the best algorithm showed a 24.6% increase in sensitivity. When these sensitivity calculations were stratified by mean count for each facility-syndrome (data not shown), we found that the modifications increased sensitivity in all strata of the DoD data; for the hospital ED data, the rate method reduced sensitivity by 1.0% in the 8 to <10 count category and by 0.5% in the 10 to <20 count category, but increased sensitivity in other categories and overall.

**Table 3:** Sensitivity for detection of additional counts, by method and dataset, for selected BioSense data used in algorithm modification study*

Minimum SD	Stratified baseline	Baseline duration, d	Sensitivity				
			Department of Defense			Hospital emergency department	
			Count	Rate		Count	Rate
0.2	No	7	40.6†	43.9		40.2†	39.1
1.0	No	7	52.3	70.8		50.4	53.6
1.0	No	14	58.6	76.8		58.7	60.9
1.0	No	28	62.0	79.4		62.8	64.8‡
1.0	Yes	7	64.9	75.7		50.2	53.8
1.0	Yes	14	75.1	80.4		57.6	60.1
1.0	Yes	28	77.0	82.0‡		60.5	62.1

*All facility–syndrome days were included in calculations. The number of additional counts varied according to categories of average count for each facility–syndrome (0.5–<2, 2–<4, 4–<6, 6–<8, 8–<10, 10–<20, 20–<40, and >40) to produce 40% sensitivity for the initial method. For the Department of Defense, the additional counts were 5.0, 9.1, 11.7, 13.6, 16.0, 20.9, 30.4, and 40.0 for the average count categories, respectively. For the hospital emergency departments, the additional counts were 4.3, 6.3, 8.2, 9.5, 10.4, 12.9, 18.7, and 28.2, respectively. †Initial method. ‡Best method for the dataset.

**Figure 2:**
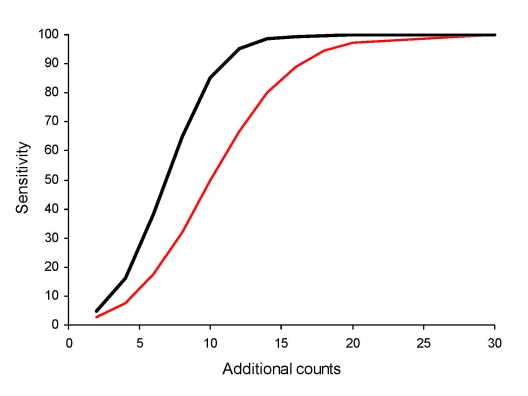
Sensitivity of detecting various numbers of additional counts, by using initial versus best algorithms for hospital emergency department chief complaint data, for selected BioSense data. Red line shows the initial algorithm (minimum SD = 0.2, 7-day baseline, count method, unstratified baseline), and black line shows the best algorithm (minimum SD = 1.0, 28-day baseline, rate method, unstratified baseline).

When we limited analysis to ED data with a mean count of 4 to <6 per day and explored sensitivity for detecting varying numbers of additional counts (Figure 2), we found, as expected, that as the number of additional counts increased, sensitivity increased. The difference between the initial and best algorithms was highest when sensitivity was ≈50% for the initial algorithm. That is, for 10 additional counts, sensitivity was 49.8% for the initial algorithm and 85.3% for the best algorithm, an improvement of 35.5%. However, if the initial C2 algorithm had either low or high sensitivity, the modifications had little effect.

**Figure 3:**
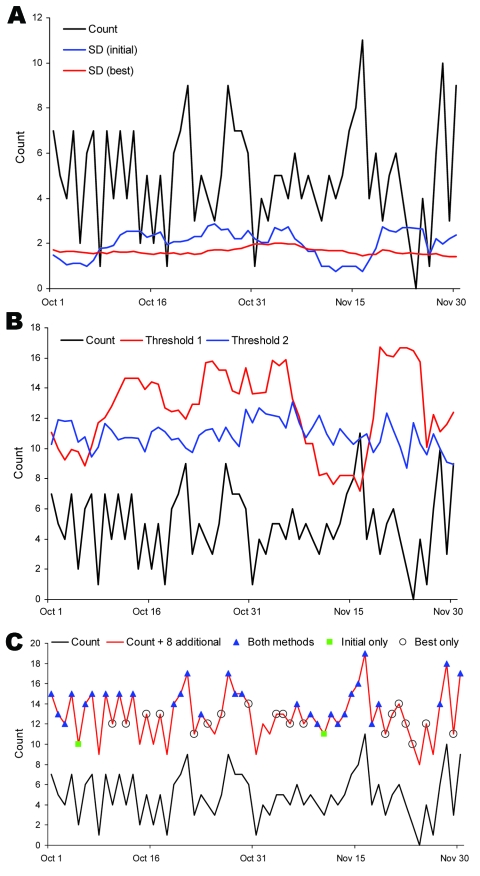
Comparison of initial versus best algorithms for analysis of fever syndrome data at an example emergency department, October–November 2006. A) SD comparison. Count, fever syndrome counts; SD (initial), SD by using initial algorithm (minimum SD = 0.2, 7-day baseline, count method, unstratified baseline); SD (best), SD by using best algorithm (minimum SD = 1.0, 28-day baseline, rate method, unstratified baseline). B) Count threshold comparison. Count, fever syndrome counts; threshold 1, minimum count needed to trigger an alert by using initial method; threshold 2, minimum count needed to trigger an alert by using best method (for the best algorithm, which accounts for rate, 8 counts were added to total visits for calculating the threshold). C) Detection of 8 additional counts. Count, daily fever syndrome counts; count + 8, daily count plus 8 counts; both methods, 30 days with the additional counts detected by both the initial and best methods; initial only, 2 days with the additional counts detected by using initial method only; and best only, 19 days with additional counts detected by using best method only.

As an example, we analyzed fever syndrome data from 1 ED. The mean count was 4.9 per day, and the 99th percentile threshold values were 3.86 SDs for the initial and 3.55 for the best algorithm. Over 632 days, the sensitivity for detecting 8 additional counts was 47.2% for the initial and 70.9% for the best algorithm (23.7% difference). Data for a 2-month period showed that the calculated SD (Figure 3, panel A) and the threshold value (i.e., count needed to trigger an alert; Figure 3, panel B) varied substantially for the initial algorithm but were comparatively stable for the best algorithm. During the 2-month period, 8 additional counts would be detected by initial and best algorithms on 30 days, by only the initial algorithm on 2 days, and by only the best algorithm on 19 days; neither algorithm detected the additional counts on 10 days (Figure 3, panel C).

## Discussion

Our results demonstrate that simple modifications of the widely used C2 algorithm can substantially improve the ability to accurately recognize 1-day increases in disease syndrome activity. Depending on the dataset, mean count in the data, and the number of additional counts added, the enhanced methods may increase sensitivity by 20%–40%. These improvements were achieved without an increase in the alert rate, which was held constant at 1% for all methods. Although we chose a 1% alert rate for testing purposes, in practice, it is useful to vary the alert rate to fit the circumstances, and the BioSense application enables the alert rate to be varied between 0.1% and 2%. Regardless of the alert rate used, the modified methods have higher sensitivity. For the DoD and hospital ED datasets, sensitivity was improved by using a higher minimum SD of 1.0, a longer baseline duration of 28 days, and adjusting for total visits. Stratifying baseline days into weekdays versus weekends/holidays increased sensitivity in the DoD data, which has a strong day-of-week effect, but modestly decreased sensitivity in the hospital ED data, which does not have such an effect. Thus, the best analytic methods depend on dataset characteristics, especially the day-of-week effect, and could be varied by manual or automated selection. These findings can be used to improve both early event detection and situation awareness because accurate recognition of unusually high counts is needed for both uses.

These modifications were apparently effective for the following reasons. Accounting for total visits to the facility (i.e., rate method) produces a more accurate expected value and lower residuals (Table 2). Although number of total visits is not the ideal denominator, in general it is better than no denominator at all. An advantage of the rate method is that calculations may be made when only partial data for a given day are available. However, adjusting for total visits may reduce sensitivity slightly in some subgroups, as we found for the hospital ED data when the mean count was 8 to <20. Stratification by weekday versus weekend day improves expected value calculations when a substantial day-of-week effect exists, such as in the DoD data. When such an effect is not present, stratification causes days further from the index day to be used in the baseline period, therefore producing slightly less accurate expected values. Longer baseline durations have no effect on the accuracy of expected value calculation and improve sensitivity by producing more accurate and stable SD values. Using a higher minimum SD avoids nuisance alerts that may be prompted by small fluctuations in the daily visit count. This method also changes the distribution of test statistic values, which results in a lower 99th percentile cutoff value, which increases sensitivity for detecting moderate-to-high numbers of added counts. Using a higher minimum SD is beneficial if disease indicators with low and high counts are analyzed; an alternate approach is to use different methods for low- versus high-count data.

The issues focused on by our suggested modifications may alternately be addressed by various sophisticated mathematical modeling approaches. However, health departments, which are generally limited in resources and in analysis expertise, may resist use of decision-support methods that are expensive, difficult to implement, or not transparent to human data monitors. For example, sophisticated Serfling-type regression models have long been used by CDC for tracking the progress of influenza season (*13**,**14*) and have been used to analyze selected data in the BioSense system. However, these models have both strengths and weaknesses and have not been widely embraced for daily disease surveillance. Even if the expertise and hardware capability for applying them were made available to local health departments, many time series are unsuitable for this approach. We present simple and easily understood and implemented enhancements to C2 to extend its applicability and improve its performance. These enhancements may be applicable to other control chart-based algorithms as well.

Automated surveillance systems based on chief complaints and diagnoses have a number of uses: providing assistance in data collection; monitoring seasonal influenza (*15*); monitoring total ED visits during a crisis; and monitoring simple surrogates of infectious diseases, injuries, and chronic diseases during large outbreaks or disasters (*16*). The utility of these systems has not been demonstrated for monitoring small- or intermediate-sized outbreaks or illnesses defined primarily by laboratory testing. Even when using these suggested modifications, sensitivity for detecting additional counts at the facility level remains modest. However, the utility of automated biosurveillance will be expanded with the availability of better population coverage and more specific data, the use of multiple data types in combination, and improved detection algorithms, such as those proposed here.

The limitations of this study include using only data with a mean count >0.5 per day; analyses of sparser data might show different results. We studied only facility-level aggregation of data, selected patient types (e.g., hospital inpatients were not studied), selected data types (e.g., ED diagnoses were not studied), and broadly defined syndromes (the more granular subsyndromes, which are likely to yield lower counts, were not studied). Although we evaluated only a simple time-series detection method, optimizing performance of simple methods is useful before they can be meaningfully compared with more sophisticated methods, such as regression. Also, we studied effects of additional counts on single days rather than multiday outbreak effects; however, because the C2 algorithm considers data from only 1 day at a time, this is a reasonable initial approach. These results must be confirmed by trials of multiday signal injection and performance evaluated for multiple subgroups (e.g., syndrome, day of week, season). We adopted the approach of evaluating sensitivity at a fixed 1% alert rate defined empirically for each algorithm and dataset, as used by Jackson et al. (*12*). Our approach is in accord with a recent review that recommended basing alert thresholds on empirical data rather than on classical statistical theory (*17*). A major strength of the study is that BioSense is a national system that provided access to 2 major datasets with differing characteristics and to data from hundreds of facilities in many states. The length, geographic spread, and syndrome variation of the study datasets lend weight to the results.

The field of electronic biosurveillance is in its infancy and is rapidly changing. Early work focused on attempts to detect outbreaks (early event detection) by using broadly defined syndromes (e.g., respiratory syndrome) based on chief complaints and diagnoses. Emphasis has recently shifted to monitoring for ongoing outbreaks (situational awareness) and for specific disease indicators (e.g., cough, dyspnea) called subsyndromes. The field is now beginning to develop methods for case-based surveillance (i.e., automated application of a formal case definition using computerized data) (*18*). Each data type and disease indicator may have unique characteristics that require modifications of standard data analysis methods. However, because the adaptation of time-series methods to recognize outbreaks will be an ongoing need, the enhanced methods identified by this study are likely to have lasting usefulness.
